# On Spirit of Turpentine in the Yellow Fever

**Published:** 1806-01

**Authors:** 


### On Spirit of Turpentine in the Yellow Fever.

THERE is a period in this formidable disease, when suddenly the forces of the patient are entirely exhausted, and a disorganization of the solids, together with a decomposition of the fluids take place. Complete jaundice, the absolute insensibility of the stomach, singultus, vomiting of a black, fœtid matter, and a trembling pulse, are the dreadful diagnostic signs of this state. The medicines hitherto used for it, have seldom had the desired effect; Dr. Holst therefore recommends the Spirit of Turpentine. He has had an opportunity of administering this medicine internally as well as externally, in some of the worst species of typhus, and he thinks with better effect than the other medicines generally used, seemed to produce. In particular, the turpentine revives the function of the liver, and also promotes the action of the organs of digestion. In a chemical respect the spirit of turpentine consists of hydrogen and carbon, which elements are slightly combined together, and easily to be separated; the spirit therefore enters into many combinations, and penetrates the whole frame; hence the great affinity with the napthæ. It may be applied internally, and likewise in injections and frictions; and seems to deserve the attention of the profession, who have an opportunity of submitting theoretical reasoning to the test of experience.

